# Clinical and Pathological Findings of a Fatal Systemic Capillary Leak Syndrome (Clarkson Disease): A Case Report

**DOI:** 10.1097/MD.0000000000000591

**Published:** 2015-03-06

**Authors:** Andrea Zancanaro, Francesco Serafini, Giuseppe Fantin, Bruno Murer, Marco Cicardi, Luca Bonanni, Michele Dalla Vestra, Mauro Scanferlato, Giovanni Mazzanti, Fabio Presotto

**Affiliations:** From the Internal Medicine Unit (AZ, FS, GF, LB, MDV, FP); Pathology Unit (BM), Angelo General Hospital, Venice; Internal Medicine Unit (MC), Luigi Sacco General Hospital, University of Milan; and Internal Medicine Unit (MS, GM), San Donà di Piave General Hospital, Venice, Italy.

## Abstract

Systemic capillary leak syndrome (SCLS) is a rare disorder with episodes of hypotension, hypoalbuminemia, and hemoconcentration. During attacks endothelial hyperpermeability results in leakage of plasma proteins into the interstitial space. Attacks vary in severity and may be lethal.

A 49-year-old previously healthy man was admitted to hospital for hypovolemic shock, anasarca with pleuropericardial effusion, muscle fatigue, and oliguria occurring after a flu-like syndrome. Laboratory data showed an increase in hematocrit (65%), leucocytes (24.590 μ/L), creatinine (2.5 mg/dL), creatine phosphokinase (10.000 U/L), and a decrease in serum albumin (17 g/L) without proteinuria. Immunoglobulins of class G/λ monoclonal gammopathy were detected (1.3 g/L). The initial suspicions addressed to a protein-loosing syndrome or to an effort-related rhabdomyolysis. Initial therapy was based on steroids, albumin, and high molecular weight plasma expanders (hydroxyethyl starch). Because of high hematocrit, phlebotomy was also performed. The patient had complete clinical remission and a diagnosis of SCLS was finally made. He received prophylactic therapy with verapamil and theophylline that was self-stopped for intolerance (hypotension and tachycardia). He had a new crisis 2 days after a physical effort, and was admitted in intensive care unit. The patient died for severe hypovolemic shock with multiorgan failure and sudden cardiac arrest 15 hours after hospital admission. Postmortem investigation revealed massive interstitial edema of main organs with myocardial hyperacute ischemia.

Studies on SCLS are limited for the rarity of the disease and its unpredictable course. Both prophylactic and acute crisis treatments are empirical and optimal management of severe attacks is still lacking.

## INTRODUCTION

Systemic capillary leak syndrome (SCLS), also known as Clarkson disease, is a rare disorder characterized by episodes of severe hypotension, hypoalbuminemia, and hemoconcentration.^1^ A monoclonal gammopathy of unknown significance, typically of the immunoglobulins of class G (IgG) class, is present in most of the SCLS adult cases.^2,3^ During acute crisis of SCLS, profound derangement of the vascular endothelium results in leakage of plasma and proteins into the interstitial compartment. Episodes differ in severity and frequency and may be life-threatening. SCLS was first described about 50 years ago, and is variably referred to as Clarkson disease or syndrome.^1^ Approximately 250 cases of SCLS have been reported worldwide since 1960.^3–5^ The 5-year survival rate is about 75%, and deaths are most commonly related to acute SCLS events. These have been diagnosed primarily in middle-aged adults, although cases in children have also been reported.^5,6^ We describe here a case of fatal SCLS associated with physical effort.

## CASE PRESENTATION

A 49-year-old Italian man was admitted to hospital in August 2012 for suspected gastroenteritis. He was a pharmaceutical representative practicing sports during free time. The patient had a previous diagnosis of microurolithiasis with calcific prostatitis, and his relevant clinical history had began in April 2012, 2 days after a marathon (a long-distance running with of 42 km) with a flu-like syndrome characterized by fever (37.5°C), muscle weakness, and dyspnoea. On that occasion, he went to the emergency department (ED) where his blood tests revealed high hemoglobin levels (18.9 g/dL, hematocrit 54%), with mild increase in serum creatinine (1.3 mg/dL). Symptoms and blood test were ascribed to dehydratation secondary to the prolonged physical effort. Assumption of eritropoietin as anabolic substance and/or diuretics was also suspected but firmly denied by the patient. He therefore underwent hydratation and phlebotomy. He was discharged and then addressed to hematological investigation. Primary or secondary polycythemia was excluded by the absence of mutation in Janus kinase 2 gene and normal arterial gas analysis. Clinically, the patient rapidly improved but in May 2012 he developed progressive swelling of the face and of the lower limbs within 12 hours, after apparently no preceding efforts, with body weight increase of about 10 kg. Oral furosemide (50 mg) and steroid (prednisone 25 mg) assumption given by his general practitioner for a few days was followed by prompt reduction of edema and body weight.

On 23 August 2012, he had a running of 18 km. Three days later, he complained of gastrointestinal symptoms with nausea, vomiting, abdominal pain, diarrhea, and fever (38.5°C). He therefore took oral paracetamol 500 mg thrice a day and metoclopramide for 2 days with no appreciable improvement. He went to the ED for the occurrence of deep asthenia, hypotension, and oligoanuria. Blood test showed hemoglobin levels of 22 g/dL, hematocrit 64%, and creatinine 2.7 mg/dL (Table 1). The patient was therefore admitted to the Internal Medicine ward. He presented with bilateral edema of the legs and forearms. The blood pressure was 90/50 mm Hg, the electrocardiogram (ECG) showed sinus tachycardia. Echocardiography and thoracoabdominal computed tomography were negligible. Hypotension was treated with saline solutions and dopamine infusion (4 mcg/kg/min). However, because of persistent hypotension and occurrence of increasing anasarcatic state with lower limb weakness, he was transferred to intensive care unit where he was treated with albumin infusion and phlebotomy (total amount of 600 mL). ECG showed paroxysmal atrial flutter with ST-depression on V_1–3_ derivations. Because he also developed numbness, tingling, and weakness of both his legs, Guillain–Barrè syndrome was suspected. Cerebral nuclear magnetic resonance, electromyogram, and rachicenthesis at that time were unremarkable. Heart ultrasound investigation showed no abnormalities with a normal ejection fraction (57%).

**TABLE 1:**
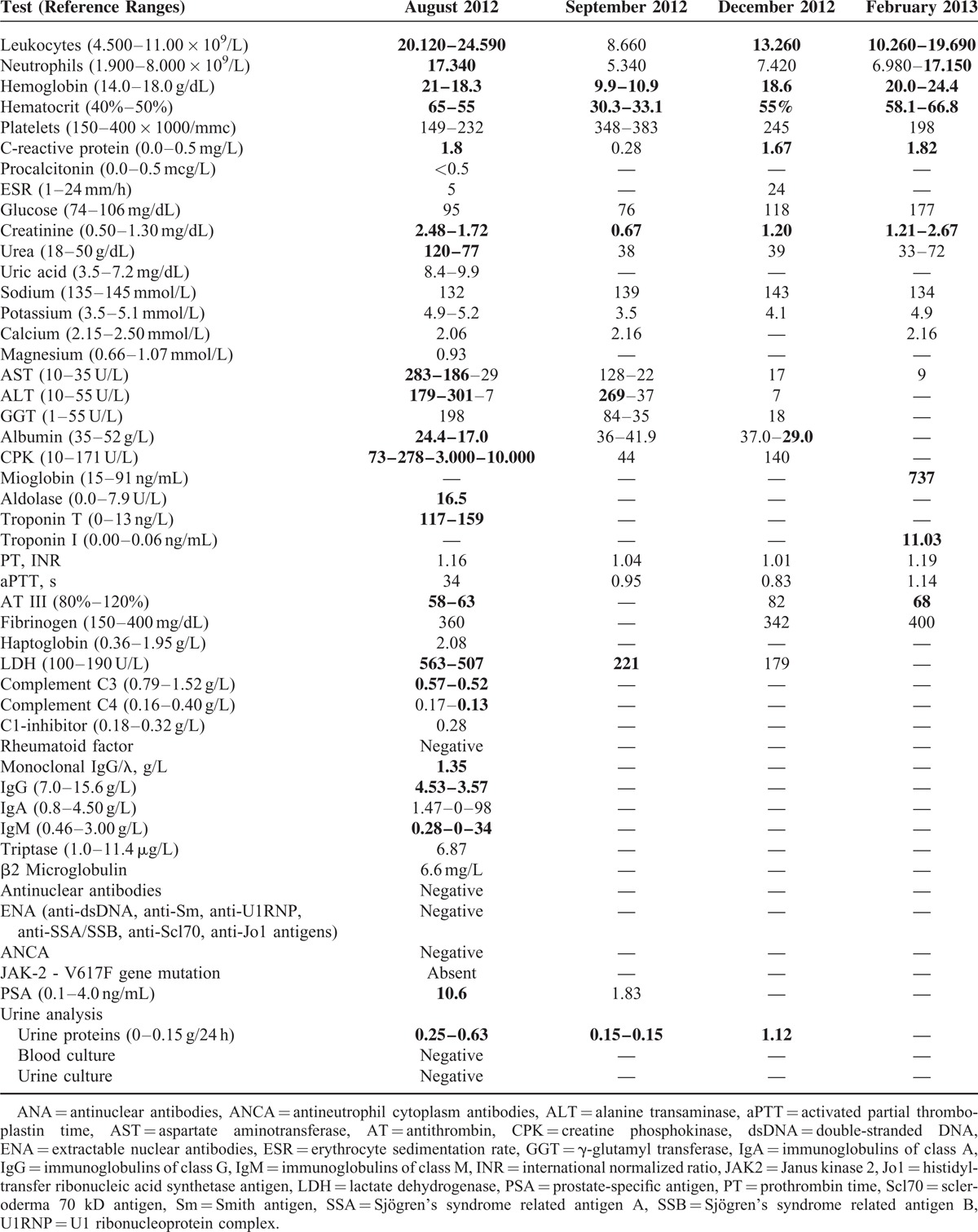
Laboratory Studies During Hospital Admissions (Abnormal Results in Bold)

The patient progressively improved and was then transferred to our Internal Medicine Unit. During hospital admission, his clinical state remained stable with the exception of a mild residual perimalleolar edema, which recovered quickly after the use of mild doses of furosemide. However, he continued to complain of symmetrical paresthesia of the calves (with sock-like distribution), and this time an electromyogram revealed axonal sensitive polyneuropathy of lower limbs. The cause of the observed hypoalbuminemia was investigated. He was well nourished and did not have any evidence of chronic inflammatory conditions, and the measured proteinuria of 0.63 g/24 h was unlikely to explain the degree of observed perimalleolar swelling. Serum tryptase and C1 esterase levels were normal. The patient was then discharged and the typical clinical presentation was recognized as SCLS. This diagnosis was confirmed after a second evaluation at another hospital center. At discharge a prophylactic therapy with verapamil 40 mg twice a day (up to 240 mg) and theophylline 375 mg every day were prescribed. However, this treatment was discontinued by the patient's own decision, because of reported intolerance for hypotension, heart palpitations, and tremor.

On December 2012, the patient complained of fever, tiredness, diarrhea, oliguria, facial and lower limb swelling, cough, and x-ray detection of right pneumonia. Blood pressure was normal. Fever and symptoms improved after a course of therapy with levofloxacin, use of plasma expanders (6% hydroxyethyl starch), and low doses of diuretics. The symptom improved within a few days.

On January 2013, after 2 days of a sustained physical exertion (ski mountaineering), the patient complained again of progressive drowsiness, oliguria, swelling of the limbs, and hypotension but no fever, cough, or diarrhea. He was therefore admitted to intensive care unit and unsuccessfully treated with plasma expanders, steroids, and dopamine infusion. Fifteen hours later he died with a multiorgan dysfunction syndrome and sudden cardiac arrest despite resuscitative efforts.

**FIGURE 1:**
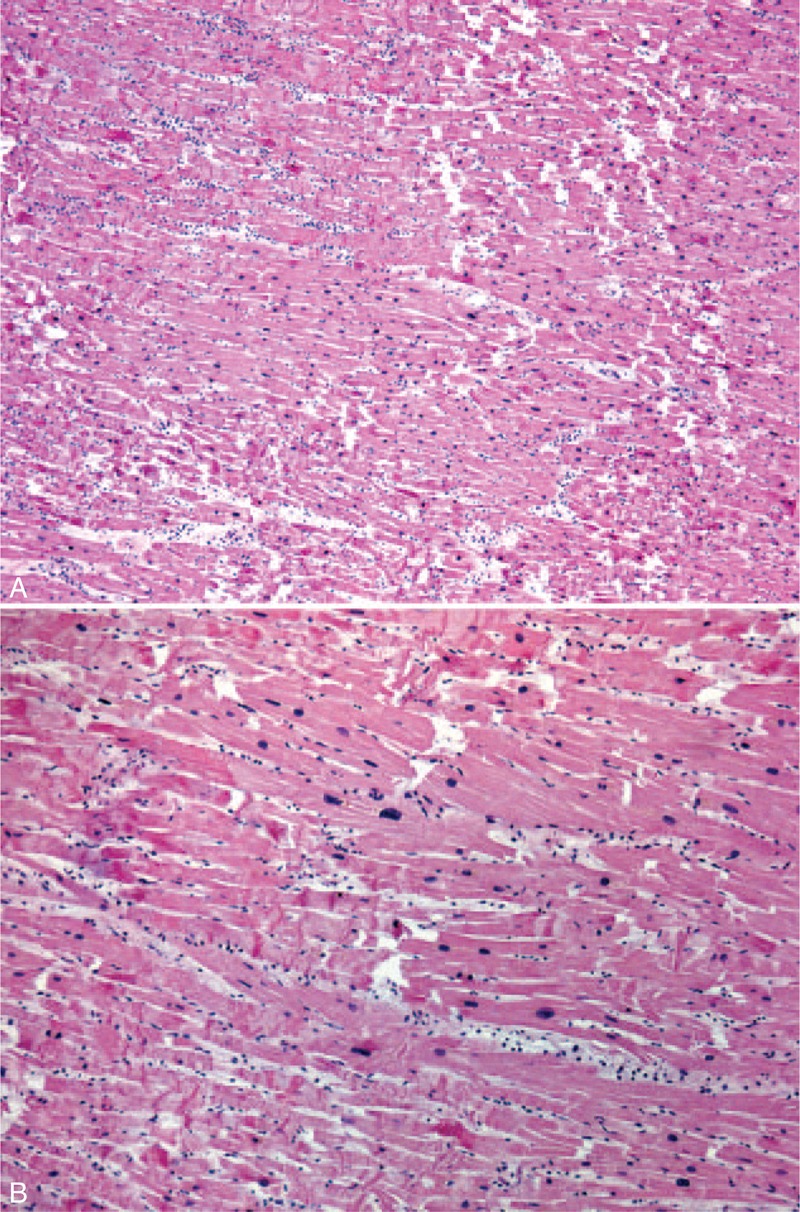
(A and B) Early phase of acute myocardial infarction. Images show diffuse and prominent contraction bands of myocardial fibers and polymorphonucleocytes in the interstitium (hematoxylin and eosin stain).

At necropsy, the pathological findings were characterized by moderate bilateral pleural and pericardial effusion and by a diffuse interstitial edema involving lungs, liver and kidney, and soft tissues. The most important finding was a prominent myocardial edema associated with contraction bands and hydropic swelling of myocardial fibers and early extravasation of polymorphonucleocytes producing the morphological pattern of acute myocardial infarction in its very early phase. Main coronary arteries and intramyocardial vessels were normal (Figure 1A and B).

## DISCUSSION

Idiopathic SCLS is a rare disorder characterized by intermittent attacks of diffuse microvascular hyperpermeability. During acute episodes, the seepage of fluids and proteins from the intravascular space results in diffuse or segmental edema, hemoconcentration, and hypotension.^1,3^ Attacks greatly vary in severity and may be lethal, but the final cause of death still remains unclear. We here reported a fatal case of SCLS due to sudden cardiac arrest in whom, to the best of our knowledge, postmortem examination was performed for the first time and myocardial histology has been described.

The original description of Clarkson in 1960 in New York Hospital reported an otherwise healthy 34-year-old white woman of Italian descent who on repeated occasions had massive swelling of the face, arms, and legs, with progressively decreasing, and finally unmeasurable blood pressure with irreversible hypovolemic shock.^1^ Since then, <300 cases have been reported worldwide.^7^

The pathophysiology of SCLS remains poorly defined. Several studies have found that the majority of patients in series and case reports had monoclonal proteins.^3,8,9^ Monoclonal IgG gammopathy was observed in 25 of a case series of 28 patients (89%) from a European registry.^3^ The paraproteins are most often of the IgG class with κ light chains typically identified in the serum.^10,11^ Paraprotein is probably not directly involved for disrupting the vascular endothelial barrier, because studies in which healthy endothelial cells were exposed in vitro to paraproteins of patients showed no effects.^12^ Moreover, the amount of monoclonal immunoglobulin produced is small and not fluctuating, whereas symptoms usually are. Xie et al^12^ recently showed that circulating permeability factors in SCLS serum induce endothelial permeability in vitro by disrupting endothelial adherens junctions and cause cell retraction without inducing cell death. This reaction occurred even when the immunoglobulins’ fraction of the serum were removed and prevented in the presence of intravenous immunoglobulin (IVIG). They propose that vascular endothelial growth factor and angiopoietin 2 may be the factors involved, as both were found to be elevated in episodic SCLS sera but not in remission sera.^12^

In our patient, the association between progressive edema, severe hypotension with multiple organ dysfunction, *inspissatio sanguinis* with elevated hematocrit, decreased albumin levels, rhabdomyolysis, and monoclonal gammopathy finally addressed to the diagnosis. The main trigger for acute episode was physical exercise, although a concomitant gastroenteric infection might not be excluded on one occasion. In a series of 28 patients with SCLS, Gousseff et al^3^ found that the potential triggers for attacks were infections (74%), mainly of the upper respiratory tract, and sustained physical effort in a minority of patients (7%).

A protein-loosing syndrome (enteric and/or renal) was first suspected in our patient, but this hypothesis was not confirmed afterwards. The slight proteinuria first detected improved spontaneously, being a frequent finding in physical efforts. Moreover, rhabdomyolysis was first wrongly ascribed to the strenuous exercise and/or to paracetamol therapy,^13^ but after a more detailed clinical history in our patient creatine phosphokinase and myoglobin elevation were found to occur after the development of the anasarcatic state and not before. Acute rhabdomyolysis was therefore a consequence of the plasma leakage and development of acute compartment syndrome. This is a serious complication of SCLS reported during the extravasation and recovery phase. It is caused by the escape of fluids into the muscular compartment of the extremities that increases tissue pressures inside this compartment, and fluid resuscitation may exacerbate this complication. Rhabdomyolysis following the compartment syndrome contributes to the onset of acute kidney insufficiency, as ultimately occurred in our patient.^14–16^ Investigation of the hypotensive episode led to the exclusion of adrenal mineralocorticoid and corticosteroid insufficiencies. A normal serum tryptase excluded a systemic mastocytosis or anaphylaxis, whereas a normal C1-esterase level excluded angioedema.

The histological findings, exceptionally reported in these patients, are not specific and reflect the leakage of fluids into different compartments with an anasarcatic state. In this patient, we found a significant involvement of the myocardium characterized by a remarkable interstitial edema with evidence of acute myocardial ischemia in its early stage (Figure 1A and B). This represents a rare complication in SCLS and may be associated with major arrhythmias that may be the final cause of death in our patient. As expected, we did not have morphological evidences of coronary vessel lesions and secondary ischemic abnormalities.

Because the etiology of SCLS is unknown, prophylactic treatment remains no evidence-based and mainly empiric. Many different treatments have been attempted, such as β2-agonist, *Gingko biloba* extract, IVIG, and leukotriene receptor antagonists.^3^ Recent research demonstrates IVIG to be a promising alternative treatment strategy for SCLS, functioning possibly through their anticytokine properties and immunomodulating effects.^17,18^ Xie et al^18^ reported that IVIG prophylaxis (1–2 g/kg/mo) performed in 18 patients induced a dramatic reduction in the number of episodes of hypovolemic shock and edema in patients with classic acute SCLS. The median yearly attack frequency was 2.6 per patient prior to IVIG therapy and nil per patient following initiation of IVIG prophylaxis, with minimal side effects.

## CONCLUSIONS

Studies on SCLS are limited for the rarity of the disease and its unpredictable course. Even if the diagnosis of SCLS is achieved, its treatment is largely empirical, and optimal management of severe attacks in intensive care units is still lacking and should be a future goal. IVIG may be considered a frontline therapy for subjects with a clear-cut diagnosis of SCLS and recurrent attacks.

### Main Take Home Lessons of This Case Report

SCLS is characterized by:

1. recurrent episodes of distributive and hypovolemic shock,
2. generalized edema due to fluid extravasation, that may lead to compartment syndrome,
3. severe hemoconcentration with high hematocrit,
4. hypoalbuminemia, and
5. unresponsiveness to fluid administration.

Acute crisis is life-threatening, but periods between attacks are usually asymptomatic.

A monoclonal IgG gammopathy is frequent in adult patients.

The treatment of acute crisis is largely empirical, and optimal management of severe attacks is still lacking.

Prophylactic therapy with high doses of IVIG seems helpful in the prevention of acute crisis.

## ACKNOWLEDGMENTS

We greatly thank Dr Monica Geremia and Dr Silvia Zampieri of Intensive Care Unit for their passionate contribution to this clinical case.
